# Case Report: Low-intensity transcranial focused ultrasound stimulation improves social interaction and stereotyped behavior in a boy with autism spectrum disorder

**DOI:** 10.3389/fpsyt.2025.1606300

**Published:** 2025-07-22

**Authors:** Shu Cheng, Xuan Xu, Chao Shan Yan, Meng Chai Mao, Kai Xuan Luo, Xiao Fan Zhang, Qi Hui Liang, Xiao Jing Long, Li Juan Ao, Mo Xian Chen

**Affiliations:** ^1^ School of Rehabilitation, Kunming Medical University, Kunming, China; ^2^ Department of Rehabilitation Medicine, Wuhan University of Science and Technology Affiliated Wuhan Resources and Wisco General Hospital, Wuhan, China; ^3^ School of Medicine, Tongji University, Shanghai, China; ^4^ Shanghai Yangzhi Rehabilitation Hospital (Shanghai Sunshine Rehabilitation Center), Tongji University School of Medicine, Shanghai, China; ^5^ Engineering Research Center of Traditional Chinese Medicine Intelligent Rehabilitation, Ministry of Education, Shanghai, China; ^6^ School of Rehabilitation Science, Shanghai University of Traditional Chinese Medicine, Shanghai, China; ^7^ Department of Psychiatry, Tongji Hospital of Huazhong University of Science and Technology (HUST), Shanghai, China; ^8^ Department of Rehabilitation Medicine, Yunnan Rehabilitation Center for The Disabled, Kunming, China; ^9^ Shenzhen Institute of Advance Technology, Chinese Academy of Sciences, Shenzhen, China

**Keywords:** autism spectrum disorder, transcranial focused ultrasound stimulation, social interaction, functional near-infrared spectroscopy, dorsolateral prefrontal cortex

## Abstract

**Objectives:**

Presently, no biomedical therapies are available that specifically address the core symptoms of autism spectrum disorders. Given the evidence of cortical malfunction in ASD, low-intensity transcranial focused ultrasound stimulation has been discussed as a prospective therapeutic technique.

**Methods:**

We describe the application of transcranial focused ultrasound to the left dorsolateral prefrontal cortex in a boy with ASD, which was applied for 30 minutes each consecutive weekday for four weeks (20 sessions in total). Social interaction, stereotyped behavior and language were assessed by scales before the first transcranial focused ultrasound session, immediately after 2 and 4 weeks of treatment. Besides, functional near-infrared spectroscopy was used to detect functional connections between regions of interest and the whole brain in individuals with ASD.

**Results:**

Scale assessments revealed several improvements in social and stereotypical behavior after low-intensity transcranial focused ultrasound. The results of functional near-infrared spectroscopy indicated increasing functional connections between the SM1 and other cortical regions as well as the whole brain, which accounted for the outcomes evaluated by the scale.

**Conclusions:**

Low-intensity transcranial focused ultrasound in ASD potentially rectified cortical dysfunction, thereby presenting a novel pathway for the advancement of biomedical interventions targeting the impaired social and stereotypical behaviors in ASD.

## Introduction

Autism spectrum disorder (ASD) represents a prevalent neurodevelopmental disorder characterized by manifestations of impaired social communication and the presence of stereotyped behavior (1). Despite the majority of individuals with ASD experiencing long-term impairments in psychosocial functioning, no approved biological intervention targeting the core symptoms of ASD has been available owing to the uncertain etiology (2).

The prefrontal cortex, related to language production and social skills, peaks in synaptogenesis and plasticity between 1 and 3 years during development consistent with the altered cortical development in ASD (3). The specific pathology of synaptic maturation and plasticity during the development of ASD leads to an imbalance between excitation and inhibition (E/I ratio), especially a disproportionately high level of excitation (4). The hypothesis of minicolumnar abnormalities also suggests that cortical inhibition is deficient due to the reduced GABAergic inhibition in the minicolumn in individuals with ASD especially in the dorsolateral prefrontal cortex (DLPFC) (5, 6). Consequently, the modulation of the E/I imbalance, specifically suppressing cortical excitability, might bring about therapeutic advantages in ASD (7). Besides, neuroimaging shows that the functional connectivity in individuals with ASD was different from that in normal children (8–10).

An animal study illustrated that low-frequency repetitive transcranial magnetic stimulation (rTMS), which was an inhibitory protocol, was capable of improving autistic-like behaviors in rats by rectifying the excitation/inhibition (E/I) imbalance (11). Consistence, many studies have implemented low-frequency TMS on the left DLPFC among individuals with ASD, manifesting enhancements in social communication deficits, repetitive behaviors, emotion regulation, and adaptive function (12–16). Teris and his colleagues indicated that transcranial pulse stimulation (TPS) over the right temporoparietal junction was effective in reducing the core symptoms of autism spectrum disorder (17). However, there has still been no research on the efficacy of low-intensity transcranial focused ultrasound (tFUS) on individuals with ASD. Low-intensity tFUS is an emerging non-invasive neuromodulation technology, the basic principle of which is to utilize ultrasound waves at a specific frequency and intensity to achieve non-invasive stimulation of specific areas. The unique advantage of this technology is that it can precisely modulate the deep target area of the brain with high spatial and temporal resolution, which is expected to bring new hope for the treatment of neuropsychiatric diseases. This case described the phenotypic and neurophysiological efficacy of tFUS on a boy with ASD.

## Case presentation

A 7-year-old boy was admitted to the rehabilitation department with language impairment accompanied by poor social skills and had a diagnosis of ASD at age 4 years. The boy had a normal weight at birth and no hypoxia or jaundice. When the child was 3 years old, parents found the boy could only utter single syllables or reduplicated words, like “baba”, “mama”, “bye-bye”, “good” and communicate and play with children of the same age without motor dysfunction. However, his speech gradually decreased, and he had no communication and played with kindergarten classmates at 4 years old. He couldn’t understand and carry out instructions, like to line up his toys and put his shoes in fixed places, along with a preference for shredded potatoes and eggs. His parents took him to the local children’s hospital without abnormal discovery in the cranial Magnetic Resonance Imaging (MRI) and Electroencephalography (EEG). After a detailed assessment of ADOS-2 by a professional pediatrician, the boy was diagnosed with ASD. Internal medicine examination did not show any obvious abnormality. The muscle strength and muscle tone of the limbs were normal. His hands could hold objects, and the fine movements of the fingers were slightly poor. But the boy’s speech, socialization and self-care skills were low. After three years of speech and sensory integration training, the boy called “Mom” and “Dad” unclearly with prompts. The concerns of his parents currently included difficulties in social interactions, language disorder, and stereotyped behavior, which hindered his primary education, and he refused to take any medication.

**Figure 1:**
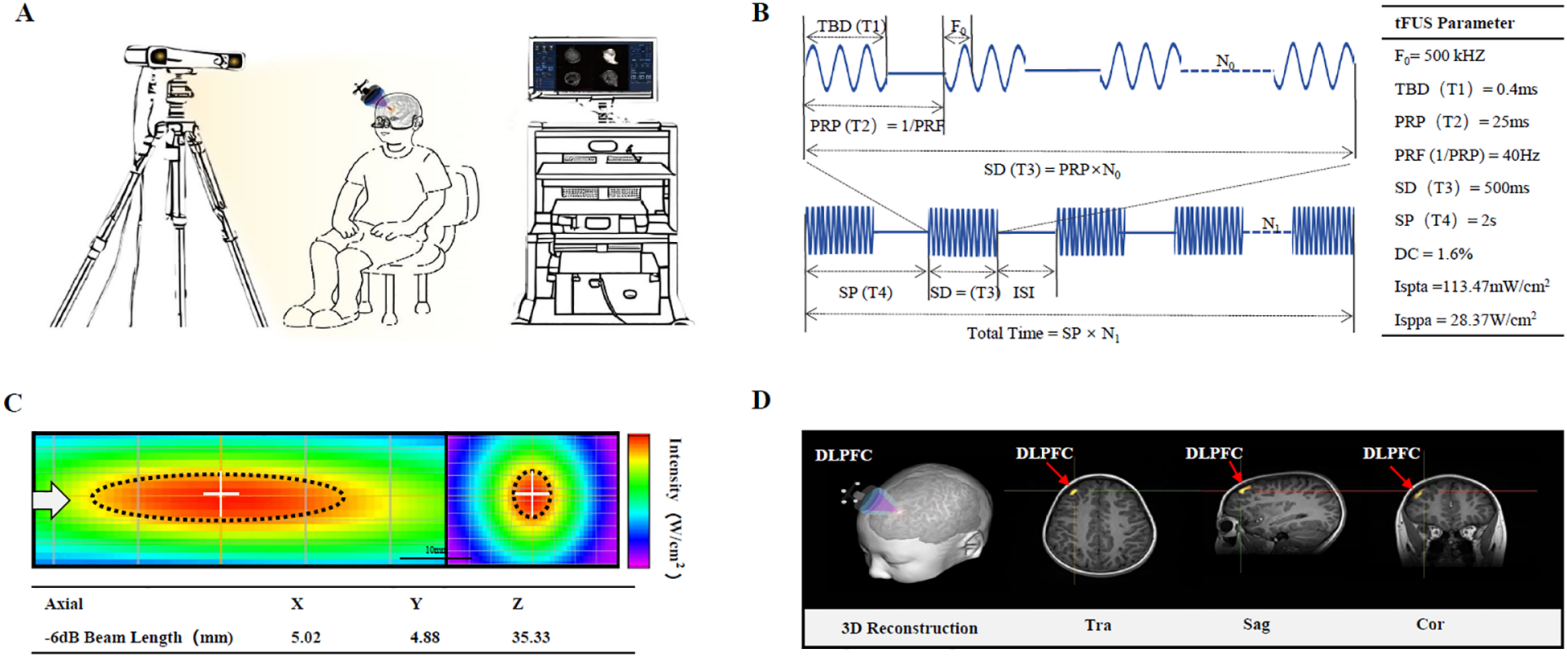
Illustration of the experimental setup for transcranial focused ultrasound (tFUS) to left dorsolateral prefrontal cortex (DLPFC) of pediatric patients with Autism. **(A)** Main device of the low-intensity tFUS system. The main device unit consists of a PC and monitor for visualization of neuroimaging and manipulation of tFUS parameters, and an infrared camera for detecting the optical tracker attached to the headgear and the tFUS transducer. **(B)** Schematic diagram of ultrasound parameters. **(C)** Acoustic intensity profile of sonication in longitudinal (YZ) plane along the sonication path and transverse (XY) planes (at the location of the white dotted line) perpendicular to the sonication is shown. The white arrow represents the direction of sonication. The full-width at half-maximum intensity profile is indicated by the dotted black ellipse and circle. **(D)** Approach for MRI-based localization of scalp site for left DLPFC.

In addition to regular rehabilitation (speech and sensory integration training lasting 3 years), the boy was administered 30 minutes of tFUS each consecutive weekday for four weeks (20 sessions in total). Transcranial ultrasonic waveforms were generated using an Ultrasound Neurostimulation System (GreenValley BrainTech Medical Technology Corporation) (**Figure 1A**). In brief, the channel output was configured to transmit a signal to actuate the designed focused ultrasound transducer. This transducer had a center frequency of 0.5 MHz, a diameter of 4.8 mm, and a focal length of approximately 24 mm, defined as the distance from the sound passing membrane to the focal point. The ultrasound pulse mode is ascertained by four elements presented on the console: the pulse width (T1), the pulse repetition period [T2, the reciprocal of which is the pulse repetition frequency (PRF)], the burst duration (T3), and the burst period (T4). The duty cycle (DC) is defined as the proportion of each pulse occupied by ultrasound cycles, expressed as a percentage value of T1/T2. The ultrasonic parameters (T1 = 400 μs, T2 = 25 ms, T3 = 500 ms, T4 = 2 s, PRF = 40 Hz, DC = 1.6%, ISPTA = 113.47mw/cm^2^, ISPPA = 28.37w/cm^2^) we used had been shown to inhibit MEP amplitudes by measuring single pulsed TMS (**Figure 1B**). The acoustic intensity profile of sonication in the longitudinal (YZ) plane along the sonication path and transverse (XY) planes (at the location of the white dotted line) perpendicular to the sonication are shown in **Figure 1C**.

Focus ultrasound stimulation navigation and guidance: the boy sat comfortably in a chair without constraints on his head. He needed to refrain from any head movements during the registration and the tFUS treatment period. T1-weighted MPRAGE (TR = 2530 ms, TE = 2.98 ms, TI = 1100 ms, Voxel size = 1.0 × 1.0 × 1.0 mm^3^) MRI was obtained to acquire the anatomical details of each participant. Subsequently, the obtained MRI data were imported into the built-in infrared image-guided FUS navigation software system. The stimulation target area (left DLPFC) was demarcated based on the anatomical MRI image. The focus position (**Figure 1D**, yellow sector) and the location of the FUS path (**Figure 1D**, red spindle) relative to the target point were presented and updated in real time on the monitor. The operator manually modified the position and spatial orientation of the transducer to align the FUS focus with the target area. The incident sound beam was required to be as orthogonal to the cranial curvature as feasible, and ultrasonic gel should be pre-applied to expel the air between the transducer and the scalp.

The boy could undergo every treatment session and reported no discomfort, such as headache, fatigue, or muscle twitching. The boy was assessed using standardized measures of autism symptoms, including the Childhood Autism Rating Scale (CARS) (18), Autism Behavior Checklist (ABC) (19), Autism Treatment Evaluation Checklist (ATEC) (20), Social Responsiveness Scale (SRS) (21) and Repetitive Behavior Scale-Revised (RBS-R) (22) before treatment, immediately after 2 and 4 weeks treatment. The scores of five assessments are directly proportional to the condition of the ASD children. The higher the score, the more severe the core symptoms are and the worse the treatment effect. Scale assessment revealed after treatment, the total CARS score for the boy dropped from 43 to 30, ABC decreased from 81 to 33 (particularly social relating scores declined from 14 to 4, sensory behavior and object use dropped to 0), and ATEC fell from 56 to 38. The scores of SRS and RBS-R dropped from 109 to 39 and from 20 to 9, respectively. Measures before and after treatment revealed a lessening of symptoms. These are presented in **Table 1**.

**Table 1:** Assessments before treatment, tFUS for 2 weeks and 4 weeks.

Behavioral assessment	Before tFUS	tFUS for 2 weeks	tFUS for 4 weeks
childhood autism rating Scale (CARS)	43	35	30
Autism behavior checklist (ABC	81	44	33
Sensory behavior	19	5	0
Social relating	14	6	4
Body and object use	11	0	0
Language	21	21	17
Social and adaptive skills	16	12	12
autism treatment evaluation Checklist (ATEC)	56	52	38
Social Responsiveness Scale (SRS)	109	45	39
Repetitive Behavior Scale-Revised (RBS-R)	20	16	9

**Figure 2:**
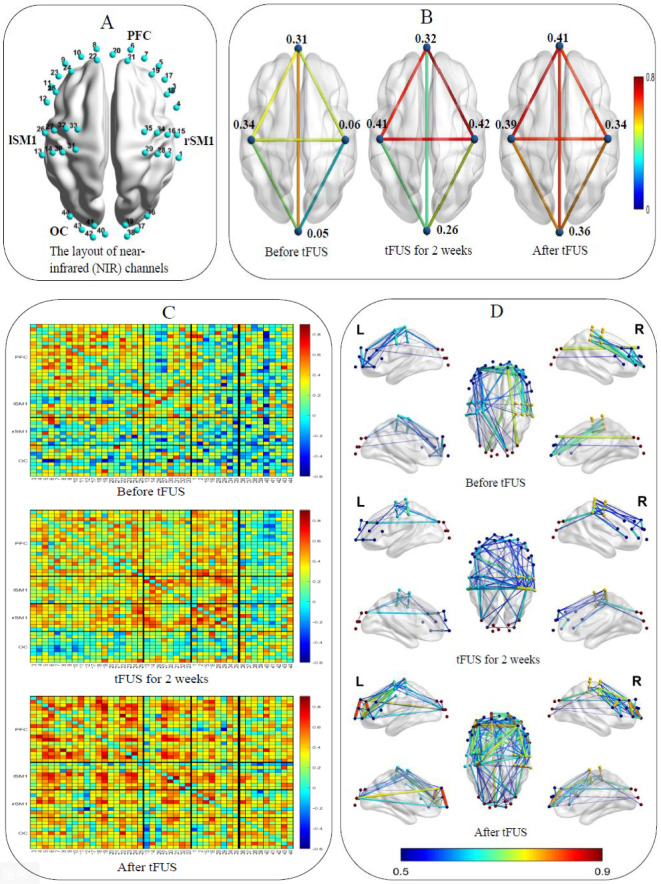
**(A)** The layout of the NIR channels in the bilateral prefrontal cortex, left and right primary sensorimotor cortex and occipital cortex. **(B)** ROI-wise functional connections between the SM1 and other cortical regions and within ROI. **(C)** Pair-wise functional connectivity in 2D. **(D)** Pair-wise functional connectivity in 3D.

Functional near-infrared spectroscopy (fNIRS) is an emerging optical neuroimaging tool that can effectively detect functional connections of different brain regions. The layout of the fNIRS channels in the bilateral prefrontal cortex (PFC), bilateral primary sensorimotor (SM1) cortex, and occipital cortex (OC) are shown in **Figure 2A**. Eyes-closed resting-state data were collected for 10 min before and after treatment. We used Homer2 and NIRS- KIT (23) based on MATLAB 2020b (The MathWorks Inc., Massachusetts) to preprocess and analyze the fNIRS data. To correct the motion artifacts, Temporal Derivative Distribution Repair algorithm was applied. Data were then lowpass filtered between 0.01 ~ 0.08 Hz with an IIR-based filter to attenuate high frequency noise. After preprocessing, functional connectivity of every paired-channels was calculated to obtain the functional connection (FC) map. For regions of interest (ROI) wise analysis, channels were divided into 4 ROIs: PFC, left SM1, right SM1and OC. Pair-wise FC within each ROI was averaged to obtain the intra-ROI FC. For inter-ROI FC, the averaged preprocessed signal was generated within each ROI firstly, and then functional connectivity was calculated between each averaged ROI data. Brain Net Viewer (23) was used to visualize FC before and after tFUS on individual with ASD. The resting-state fNIRS results found that as the treatment time prolongs, ROI-wise connections between the SM1 and other cortical regions escalated notably, while within ROI connectivity strengthened in the prefrontal cortex and visual cortex, which were involved in social interaction, language and emotion regulation (**Figure 2B**). Pair-wise functional connectivity increased to varying degrees (**Figures 2C, D**).

In addition, behavioral results show after three sessions of tFUS treatment, the child could speak a few words clearly, such as “kitten” and “puppy”. Following five sessions of tFUS treatment, his executive ability improved obviously (when coming home, and the boy help his mother remove socks and fetch slippers). The mother said that the boy’s vocabulary increased significantly, the mood was more stable than before (the number of temper tantrums decreased), and the comprehension ability improved significantly (the child could follow the instruction of not doing dangerous things) after 10 treatment sessions. After the completion of 20 tFUS treatment sessions, the child was able to dress and eat independently. His understanding and concentration abilities increased significantly (the boy could sit quietly for 30 minutes to play with toys and read picture books). Moreover, the boy sang a children’s song independently, had eye contact when being called, and the stereotyped behaviors (lining up toys and putting shoes in fixed places) disappeared.

## Discussion

Autism spectrum disorder exhibits associations with a multiplicity of environmental and genetic determinants (24). A deficiency in the understanding of the core pathologic basis of autism notably restricts the available treatment and intervention alternatives (25). This case explored the effectiveness and tolerance of 20 sessions of tFUS applied to the left DLPFC in individuals with ASD under the guidance of neuronavigation. The transcranial focused ultrasound schemes in this study were well below the FDA recommendation of a maximum ISPPA of 190 W/cm^2^ and a maximum ISPTA of 0.72 W/cm^2^.

The scores of ABC, CARS, ATEC, SRS, and RBS-R scales significantly lower compared with that before treatment demonstrated improvement in social and executive functions and reduced stereotypical behavior in the boy with ASD after a 4-week course of low-intensity tFUS to the left dorsolateral prefrontal cortex. Low-frequency rTMS interventions on the DLFPC of nineteen children with ASD found the scores of irritability, hyperactivity, and repetitive behaviors all significantly reduced. Estate and his colleagues (26) adopted bilateral low-frequency rTMS stimulation on the DLPFC of patients with ASD, and the results showed their stereotyped and aggressive behaviors were significantly improved. These results were consistent with our data. However, a few studies also indicated a lack of evidence to support the effectiveness of low-frequency non-invasive neuromodulation for autism (27) and high-frequency non-invasive neuromodulation might effectively alleviate ASD symptoms (28, 29). Variations in the frequency and duration of non-invasive neuromodulation, inclusion and exclusion criteria, sample size and assessment methods might cause these differences.

This case revealed that low-frequency tFUS improved social interaction and stereotypical behavior, alleviated irritable emotions by enhancing ROI-wise and pair-wise functional connectivity in individuals with ASD. This finding is encouraging, especially considering the absence of biomedical treatments directed at the core symptoms of ASD. Individuals with ASD exhibit significant functional disorders in cortical tissue. They have more medium- and short-range intrahemispheric connections and fewer long-range interhemispheric connections, that is, local overconnectivity and long-range underconnectivity (30, 31). An fNIRS study found that compared with normal children, the bilateral temporal lobe regions in individuals with ASD show weaker resting-state functional connectivity and weaker interhemispheric brain network. The poor social skills and restricted and repetitive patterns of interest behaviors in ASD were all associated with the weaker connectivity of brain regions (32). These research results were consistent with our study. It is worth noting that previous studies regarding the influence of neuromodulation on individuals with ASD primarily relied on the differences in scale scores, thereby exhibiting a degree of subjectivity. A more objective basis by combining with fNIRS was provided in this case.

The specific mechanism of tFUS for neuropsychiatric disorders is still unclear, and it is mainly thought to be the modulation of neural tissues by mechanical and cavitation effects. Mechanical effect refers to the tiny mechanical vibration generated when ultrasound propagates in tissues, and this vibration affects the membrane potential through neuronal mechanosensitive ion channels, realizing the conversion of mechanical signals to electrical signals, which changes neuronal excitability (33). The cavitation effect refers to the phenomenon of microbubble expansion, contraction or even bursting of gases in tissues in response to ultrasound. This process triggers changes in membrane conformation that may lead to the generation of capacitive currents, or the formation of new ion transport channels, or the activation of mechanosensitive ion channels in the membrane, thus altering their excitability (34). In addition, LIFU was found to significantly modulate the membrane potential of neurons, which in turn affects the synchronization of neural networks. Neuronal activity can be enhanced or inhibited by tFUS, then synchronization of neural electrical activity can be promoted, thus restoring neural function to a certain extent (33, 35).

## Limitation

Although this case proved to be exciting, it demands validation utilizing a placebo-controlled, double-blind, anonymized clinical trial of tFUS in individuals with ASD, which our research group is presently conducting. When writing up this case, three participants were undergoing the study and seemed to respond positively to the treatment. Another shortcoming is the lack of follow-up, requiring a long follow-up observation in future studies. The third shortcoming is that the prefrontal cortex and sensorimotor cortex were not subdivided according to Brodmann’s areas, so the functional connectivity within Brodmann’s areas was not observed. Future research can compare the functional connectivity within Brodmann’s areas.

## Data Availability

The original contributions presented in the study are included in the article/supplementary material. Further inquiries can be directed to the corresponding authors.
